# The Study of Viral RNA Diversity in Bird Samples Using De Novo Designed Multiplex Genus-Specific Primer Panels

**DOI:** 10.1155/2018/3248285

**Published:** 2018-08-12

**Authors:** Andrey A. Ayginin, Ekaterina V. Pimkina, Alina D. Matsvay, Anna S. Speranskaya, Marina V. Safonova, Ekaterina A. Blinova, Ilya V. Artyushin, Vladimir G. Dedkov, German A. Shipulin, Kamil Khafizov

**Affiliations:** ^1^Central Research Institute of Epidemiology, Moscow 111123, Russia; ^2^Moscow Institute of Physics and Technology, Dolgoprudny 141700, Russia; ^3^Lomonosov Moscow State University, Moscow 119991, Russia; ^4^Saint-Petersburg Pasteur Institute, Saint Petersburg 197101, Russia

## Abstract

Advances in the next generation sequencing (NGS) technologies have significantly increased our ability to detect new viral pathogens and systematically determine the spectrum of viruses prevalent in various biological samples. In addition, this approach has also helped in establishing the associations of viromes with many diseases. However, unlike the metagenomic studies using* 16S* rRNA for the detection of bacteria, it is impossible to create universal oligonucleotides to target all known and novel viruses, owing to their genomic diversity and variability. On the other hand, sequencing the entire genome is still expensive and has relatively low sensitivity for such applications. The existing approaches for the design of oligonucleotides for targeted enrichment are usually involved in the development of primers for the PCR-based detection of particular viral species or genera, but not for families or higher taxonomic orders. In this study, we have developed a computational pipeline for designing the oligonucleotides capable of covering a significant number of known viruses within various taxonomic orders, as well as their novel variants. We have subsequently designed a genus-specific oligonucleotide panel for targeted enrichment of viral nucleic acids in biological material and demonstrated the possibility of its application for virus detection in bird samples. We have tested our panel using a number of collected samples and have observed superior efficiency in the detection and identification of viral pathogens. Since a reliable, bioinformatics-based analytical method for the rapid identification of the sequences was crucial, an NGS-based data analysis module was developed in this study, and its functionality in the detection of novel viruses and analysis of virome diversity was demonstrated.

## 1. Introduction

An increase in globalization, climate change, and interaction with livestock animals has resulted in the emergence of novel viral pathogens or zoonoses [1], which pose a serious health problem for birds and animals and ultimately for humans. The natural reservoirs of pathogens, such as birds, bats, rodents, and bloodsucking arthropods play a significant role in the sustenance and transmission of zoonotic infections. Migratory birds warrant special attention, as their rich diversity and migratory behavior contribute to the spread of infections to considerable distances. Such migrations are strongly associated with the emergence of the epidemic and enzootic outbreaks as well as the formation and activation of natural sources of viral infections. Wild birds are widely acknowledged to be reservoirs and transmitters of pathogens responsible for emerging infectious diseases such as severe acute respiratory syndrome virus (SARSV), avian influenza virus A (H10N7), and West Nile virus (WNV), to domestic animals and humans [2, 3]. The rich biodiversity of the wild bird population may increase the risk of spread of pathogens to domesticated poultry. Understanding the viral diversity is critical for predicting future risks of transmission or possible outbreaks of viral diseases. However, identifying and monitoring the transmission of novel viruses are one of the vital requisites for responding to outbreaks. The asymptomatic carriage of viruses, which could be attributed to certain characteristics of bird metabolism and the adaptive capabilities of their immune system, provides ideal conditions for coevolution, leading to the emergence of mutant and recombinant strains of viruses. Hence, the majority of widely used molecular diagnostic methods, such as those employing polymerase chain reaction (PCR), are not sufficiently suitable for the identification of a wide variety of viruses, as the techniques are usually designed for the detection of highly conserved regions in the genomes, which limits the search to a restricted group of viral agents and prevents the identification of new viruses or viral variants. In addition, the Sanger sequencing technique, which has been a standard diagnostic tool for the detection and identification of various pathogens, provides limited sequence information at a higher cost per nucleotide base and can only be used to identify pathogens with a high titer. Moreover, a preliminary evidence of the presence of the certain viruses is required for performing the PCR, in the absence of which the process of pathogen identification could take a significant amount of time, which can be a major obstacle in the prevention and control of the infection.

DNA barcoding is a method which uses a short part of organism's genome (so-called barcode) to identify whether it belongs to a particular family, genera, or even species, by using extensive parallel sequencing technologies (or more commonly NGS [Next Generation Sequencing]). This method was initially developed for studying bacterial communities (e.g., studies on gut microbiota), but today it is widely employed for various tasks, including detection of food adulteration [4], the study of the diets of marine communities [5], and biofuel analysis [6]. Unlike other taxa, viruses lack a universally shared phylogenetic marker (such as* 16S* for bacteria,* Cytochrome C oxidase* for birds and mammals,* rbcL* and* matK* for plants, and Internal Transcribed Spacer (*ITS*) for fungi or plants), which makes it impossible to design a universal primer pair to amplify and differentiate diverse viral sequences. Furthermore, the viral taxonomy gives a better indication of the signs of diseases caused, rather than genetic similarity. This fact complicates the barcode (a short, standardized nucleotide sequence of an organism's DNA) selection, even for one genus (for example, mammarenavirus can be serologically, phylogenetically, and geographically divided into two major complexes: the Old World complex prevalent in Africa, Europe, and Asia, and the New World complex found in North and South America [7]), let alone higher taxa. However, metagenomics still allows the detection of different viral pathogens using shotgun sequencing [8]. Despite the constant reduction in the cost of DNA sequencing, this approach is still considerably expensive and is not feasible for screening a large number of samples. Also, metagenomic NGS data-sets are usually predominantly composed of host-derived sequences with only a minor fraction of pathogen sequences. Often, even an approximate fraction of pathogen content is initially unknown, making it practically impossible to estimate how many sequencing reads are needed per sample, in order to detect the pathogen in the final sequencing data file. Another common problem for most NGS-based tests is that complex multistep workflows may pose challenges in the reproducibility of results. Recently, Briese et al. [9] had developed a virome capture sequencing platform for vertebrate viruses (VirCapSeq-VERT), which consisted of ~2 million biotinylated DNA-probes for target enrichment of viral nucleic acids to increase the sensitivity of sequence-based detection and characterization of viruses. The described method allowed the identification (and possibly even sequencing the whole genome assembly of detected viruses) of a large number of viruses including the novel ones. However, the overall cost of sequencing per sample remained considerably high.

Other methods of enrichment are based on the targeted amplification of cDNA region using genus-specific primer pairs in PCR. This approach has been known for a long time [10] and has been used successfully by various researchers, both in the studies of the representatives of individual viral genera and in the analyses of the diversity of viruses in different types of biological material [11–13]. Till recently, a large number of such primers have been described [14–16]. However, most of them were designed for the detection of certain species of viruses. Moreover, it was impossible to use them in multiplex reactions due to primer-specific annealing temperatures, nonspecific amplification, and potential self-complementarity. Thus, in order to analyze one sample, it is necessary to carry out a number of PCR experiments corresponding to the number of primer pairs. The effectiveness of the study could be significantly increased by combining genus-specific PCR and NGS. In this approach, the products of different PCR assays for each sample would be pooled in one tube, purified, eluted in a minimal volume, and prepared for NGS [17]. However, this approach does not exempt the requirement of many PCRs per sample, which is a problem when a large number of samples are studied simultaneously. In addition, there are restrictions on the use of different protocols for the library preparation, particularly when the protocol includes the emulsion PCR stage, which strictly requires PCR products of the same length.

In this study, we have introduced a method for designing oligonucleotide panels for targeted enrichment of viral nucleic acids, where the main objective is to use a minimum number of primer oligonucleotides to cover the maximum number of diverse viral taxa within a single PCR reaction. We have applied this approach to design genus-specific primer pairs for targeted enrichment of cDNA from zoonotic RNA viruses and have evaluated it using several samples from birds. We have also demonstrated a considerable increase in the viral genome coverage.

## 2. Materials and Methods

### 2.1. Design of the Genus-Specific Primers Panel

This section contains the technical details of the algorithm for the design of genus-specific primer panels. To enable us to process the enrichment PCR reaction in a single tube, a number of restriction parameters were applied to the positions and structures of oligonucleotides. The availability of validated reference viral nucleic acid sequences is crucial for efficient usage of the algorithm. Although several viral reference sequence databases are publicly available [18], the medically relevant and model organisms are largely overrepresented in most of them, and the genomic diversity of circulating strains is often underrepresented. One such source is the open-source database “The Virus Pathogen Database and Analysis Resource (ViPR)” [19], which was developed back in 2011. The most important advantage of this database is the authenticity of nucleic acid sequences, as the data are curated and managed by experts in virology and bioinformatics. This source was used to retrieve the sequences of the polymerase genes of target viral genera (or other genes in cases where the sequences of polymerase genes were not available). The sequences were filtered by length (≥400 bp), quality, and intrageneric similarity and were combined to the corresponding FASTA files.

In order to create consensus sequences, nucleotide sequences within each FASTA file specific to a genus were aligned using ClustalW [20]. If a minimum of two consensus subsequences with lengths of 20 bp and a maximum of four ambiguous positions with minor nucleotide frequency ≥ 10% were not identified, the original FASTA file was iteratively clustered using CD-HIT [21], with decreasing threshold. The clusters obtained at each step were aligned independently using ClustalW to identify consensus subsequence(s) for all the subsets, which must collectively represent at least 90% of different species within the genus. Finally, at least one aligned FASTA file was obtained for every genus.

For extracting the common subsequences from multiple sequence alignments, a “sliding window” was used within a specified range of lengths. This window “slides” from the 5'- to 3'-end of the alignment with a step of one nucleotide and identifies all subsequences fulfilling the following criteria:

- proportion of ambiguous positions (*P*_*AMB*_) ≤ 20%;
- proportion of unique species, which share the subsequence and do not contain gaps (*P*_*SH*_) ≥ 50%;
- GC content of the consensus sequence (*P*_*GC*_) within 35–65% interval;
- absence of self-complementary regions;
- absence of formation of homodimers;
- absence of formation of heterodimers with previously selected oligonucleotides.

An amplicon length between two subsequences (primers) was then adjusted between 200 and 400 bp to make the panel compatible with the most popular sequencing platforms (for example, Illumina MiSeq or Ion S5 from Thermo Fisher Scientific). Two subsequences were considered to be a pair if they together covered at least 90% of species related to a target genus or cluster and shared over 90% of the species. The pairs were then filtered according to their annealing temperature (50°C ≤ T_a_ ≤ 55°C). The selected primer pairs were then aligned with the NR database using BLAST to check for their specificity. Nonspecific candidates were eliminated. The possibility of formation of heterodimers between sequences in the pair, and between the sequences and previously selected primers, was calculated using the software Primer3 [22]. Then the parameters described above were calculated and the pairs were sorted accordingly. The “best” primer pair was selected as a “genus-specific pair.”

The parameters described above were then calculated, and the pairs were sorted accordingly. The primer pair with the best fit was selected as a “genus-specific pair.”

The sequences of primer pairs for the reference viral genera, designed using the developed algorithm, are presented in Tables 1 and 2.

### 2.2. Control Samples

The ability of the developed panel to enrich the target cDNA from zoonotic RNA viruses was assessed using high titer solutions of viral RNA (concentration ranging from 10^5^ to 10^7^ copies per mL) from 24 species of viruses, belonging to 13 viral genera within 12 viral families (Table 1). Viral RNAs were sourced from the collection stored at the Central Research Institute for Epidemiology, Moscow, Russia. All viral RNAs were stored at −70°C till further use for the study. The H_2_O sterile (AmpliSens, Russia) was used as a negative control in all experiments.

The control samples cDNA was obtained by reverse transcription reaction performed on 5 *μ*L of the extracted RNA using the Reverta-L RT kit (AmpliSens; total volume of the reaction mixture is 20 *μ*L); after that 5 *μ*L of the reaction mixture containing cDNAs was further used for evaluation of the ability of the primer pair to amplify the targeted region of viruses, both in single and in multiplex PCR format.

### 2.3. Reaction Mixture and Amplification Mode

The PCR reaction mix (25 *μ*L) was prepared using 5 *μ*L of the cDNA template, 5 *μ*L of H_2_O (MilliQ, AmpliSens), 5 *μ*L of PCR mix2 FEP/FRT, 1 *μ*L of 0.2 *μ*M of each primer (in the single-plex format) or 0.08 *μ*M of each primer (total concentration of 3.84 *μ*M in the multiplex format), 2.5 *μ*L of dNTPs (1.76 mM; AmpliSens), and 0.5 *μ*L of TaqF polymerase (AmpliSens). The thermal cycling parameters were initial denaturation at 95°C for 15 min, followed by 45 cycles of 95°C for 15 s, 50°C for 120 s, 72°C for 30 s, and final extension at 72°C for 7 min.

### 2.4. Specificity of the Developed Panel

PCR products of appropriate lengths were resolved by electrophoresis on 1.5% agarose gel containing ethidium bromide. The amplicons were purified using a QIAquick PCR Purification Kit (Qiagen), following the manufacturer's instructions. The purified PCR products were then sequenced using ABI Prism 3500 sequencer (Applied Biosystems) to confirm the specificity of the reactions in a single-plex format.

### 2.5. Sample Collection

Bird samples (cloacal swabs and/or feces) were collected from the Enisei Ecological Station, Mirnoe (Russian Federation). The samples were collected from birds captured using mist-net for routine ornithological examination or from droppings left on the ground by geese at their stop-over sites. To prevent contamination, separate rubber gloves and sterile cotton swabs were used for collection of each sample. The samples were stored at the collection site for up to 30 days at 4–8°C, then for four days at room temperature during transport, and finally at 2–4°C while processing. Samples were stored in the transport medium 0.5 mL tubes for swabs containing storage solution (AmpliSens).

The samples were collected from the following species of birds: Taiga Bean Goose (*Anser fabalis johanseni*), Siberian Thrush (*Geokichla sibirica*), Song Thrush (*Turdus philomelos*), Fieldfare (*Turdus pilaris*), Redwing (*Turdus iliacus*), Black-Throated Thrush (*Turdus atrogularis*), Common Tern (*Sterna hirundo*), Green Sandpiper (*Tringa ochropus*), Common Greenshank (*Tringa nebularia*), Red-Throated Flycatcher (*Ficedula albicilla*), Temminck's Stint (*Calidris temminckii*), and Dusky Warbler (*Phylloscopus fuscatus*). No birds were harmed in this study. A total of 92 bird samples were used for further virome analysis, among which 62 samples belonged to Taiga Bean Goose and 6 to Siberian Thrush.

### 2.6. RNA Extraction and Reverse Transcription

Total RNA was extracted from 100 *μ*L of the resuspended sample with RNeasy Lipid Tissue Mini Kit (Qiagen) using robotic workstation QIAcube (Qiagen), following the manufacturer's protocol. The cDNA was obtained by reverse transcription reaction on 10 *μ*L of the extracted RNA using a Reverta-L RT kit (AmpliSens), according to the manufacturer's instructions.

### 2.7. Testing the Primer Panel with Control Samples

**Figure 1:**
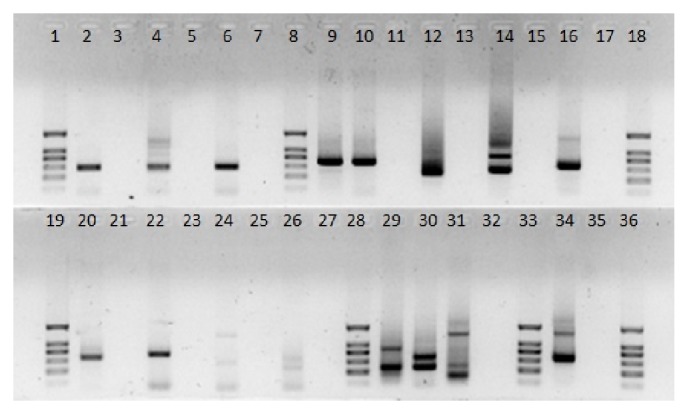
Individual primer pairs testing. (**1, 8, 18, 19, 28, 33, 36**) DNA Ladder (100 bp, 200 bp, 300 bp, 400 bp, 500 bp, 800 bp); (**2**) ZEBOV, primers ebola_f/r; (**3**) negative control for primers ebola_f/r; (**4**) MARV, primers marburg_f/r, (**5**) negative control for primers marburg_f/r; (**6**) LASV, primers mammarena_f/r, (**7**) negative control for primers mammarena_f/r; (**9**) CCHFV, primers nairo_f/r; (**10**) PRMV, primers nairo_f/r; (**11**) negative control for primers nairo_f/r; (**12**) INKV, primers orthobunya_f/r; (**13**) negative control for primers orthobunya_f/r; (**14**) DOBV, primers hanta_f/r; (**15**) negative control for primers hanta_f/r; (**16**) KEMV, primers orbi_f1/f2/r; (**17**) negative control for primers orbi_f1/f2/r; (**20**) MERS CoV, primers alphacorona_f/r; (**21**) negative control for primers alphacorona_f/r; (**22**) MERS (*Betacoronavirus*), primers betacorona_1_f/r; (**23**) negative control for primers betacorona_1_f/r; (**24**) MERS (*Betacoronavirus*), primers betacorona_2_f/r; (**25**) negative control for primers betacorona_2_f/r; (**26**) MERS (*Betacoronavirus*), primers gammacorona_f/r; (**27**) negative control for primers gammacorona_f/r; (**29**) TBEV, primers flavi_f/r; (**30**) YFV, primers flavi_f/r; (**31**) JEV, primers flavi_f/r; (**32**) negative control for primers flavi_f/r; (**34**) RABV, primers lyssa_f/r; (**35**) negative control for primers lyssa_f/r.

The primer panel was tested with a reference set of samples (Table 1). The results are presented in Figure 1. In all cases, the products of the specified range of lengths were obtained.

**Figure 2:**
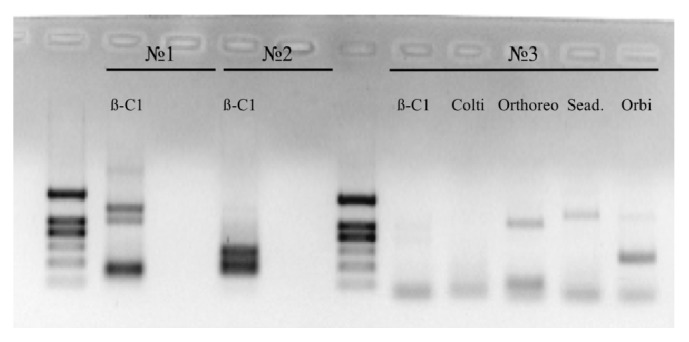
Reamplification of the multiplex PCR product; ß-C1:* Betacoronavirus*-specific primers; Colti: coltivirus-specific primers; Orthoreo:* Orthoreovirus*-specific primers; Sead.: Seadornavirus-specific primers; Orbi: orbivirus-specific primers.

The multiplex system was then tested with the same set of viruses and the PCR products were reamplified using the genus-specific primer pairs. In all cases, unspecific amplification was observed as well. However, the reamplification reactions with the genus-specific primers showed the presence of the target products. The multiplex system was first tested with three bat samples infected by several known viruses (sample N1:* Betacoronavirus*, sample N2:* Betacoronavirus*, sample N3:* Orthoreovirus*). The reamplification of the PCR products was carried out with the genus-specific primers (Figure 2). The obtained results clearly demonstrated the presence of the products with target length in all three samples.

### 2.8. Preparation and Sequencing of Ion S5 Libraries

The targeted sequences were enriched by multiplex PCR with the designed primer pool described above (Table 2). PCR products were cleaned using carboxyl-coated magnetic particles, commercially available as Sera-Mag Speed Beads (GE Healthcare). The concentrations of the fragments were measured using Qubit dsDNA HS Assay Kit with a Qubit 2.0 fluorimeter (Invitrogen).

The preparation of the amplicon libraries involved phosphorylation of the 5'-end and incorporation of barcoded adapters, followed by amplification of the final library. For this purpose, T4 Polynucleotide Kinase and T4 DNA Ligase (both from New England Biolabs, NEB) were used according to the manufacturer's protocol with slight modification. Amplification was performed using PCR-mix 2 FEP/FRT (AmpliSens).

The two total RNA libraries for Ion S5 high-throughput sequencing were prepared from total RNA of two bird samples (*B23* and* B66*). The first strand of cDNA was synthesized using random primers and Reverta-L RT kit (AmpliSens). The second strand of cDNA was prepared with NEBNext Ultra Second Strand Synthesis Module of Kit #E7530 (NEB). The double stranded DNA was fragmented by Ion Shear Plus Reagent Kit (Thermo Fisher Scientific) and libraries preparation was performed using Ion Xpress Plus Fragment Library Kit (Thermo Fisher Scientific) according to the manufacturer's guide.

The quality of final libraries was assessed on the Agilent 2100 Bioanalyzer (Agilent Genomics), employing the Agilent High Sensitivity DNA Kit (Agilent Genomics). Amplicon libraries were separated by 1.7% agarose gel electrophoresis stained with ethidium bromide, and the fragments of target lengths were cut out and purified using the MinElute Gel Extraction Kit (Qiagen). Size selection of the final total RNA libraries was done using 2% E-Gel™ SizeSelect™ II Agarose Gels (Thermo Fisher Scientific) on the E-Gel electrophoresis system (Thermo Fisher Scientific).

Sequencing was carried out on the Ion S5 platform using Ion 520/530 Kit-Chef reagent sample preparation kit and employing Ion 530 chips on the Ion Chef instrument (Thermo Fisher Scientific).

### 2.9. Bioinformatics Analysis

**Figure 3:**
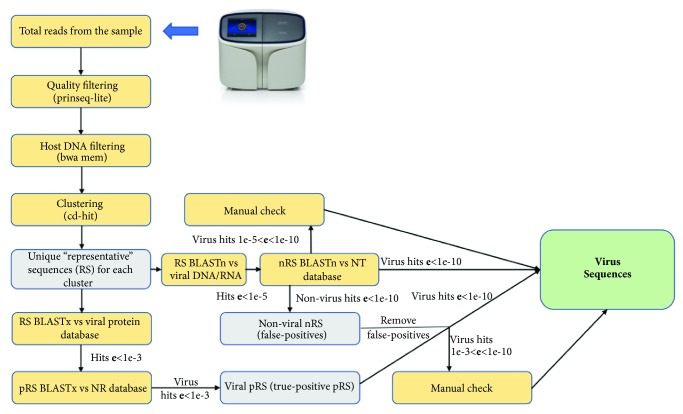
A schematic picture of the bioinformatics pipeline developed for the analysis of the NGS data in this study. Specific third-party tools that were employed are shown in parentheses.

Raw sequencing reads obtained from the platform were first filtered using the PRINSEQ-lite tool [23] to eliminate too short (<80 bp) and low-quality (min_mean < 20) fragments. The mean, median, and 10th and 90th percentiles of read lengths distributions for selected unfiltered FASTQ files are shown in the supplementary Table S1. The BWA software [24] was used then to align the filtered reads to the reference birds' genomes database. The ideally aligned reads (full read length and no mismatches) were marked as nonviral reads and were eliminated. The software CD-HIT [21] was used to reduce the redundancy, i.e., the number of reads per sample, by clustering (with a similarity threshold 90%) and selecting the representative sequences for each cluster. Briefly, the CD-HIT algorithm sorts the sequences from long to short and processes them sequentially. The first sequence is automatically classified as the first cluster representative sequence. Then each query sequence from the remaining sequences is compared to the representative sequences identified before it and is classified as redundant or representative based on whether it is similar to one of the existing representative sequences. The described filtering and clustering steps significantly reduced (by ~100 times) the computational time of the further steps. The software BLAST was then employed to first compare the representative sequences (RS) against virus-only nucleotide and protein databases that were collected by selection of virus sequences from GenBank NT and GenBank NR databases, respectively, to identify viral nucleotide candidate RS (nRS) and protein candidate RS (pRS) with E-value cutoffs of 10^–5^ and 10^–3^, respectively. Since virus-only databases are much smaller than NCBI NT and NR databases, pairwise sequence comparisons are much faster, which maximizes the speed and allows for the more efficient use of computational resources. We subsequently aligned these candidate nRS and pRS to the entire GenBank NT and NR databases, respectively, to eliminate potential false positives (sequences with higher similarity to nonviral reference sequences than to viral ones in the database) among the candidates and to select the true positives. The additional step was applied to select true positives from viral pRS: the pRS aligned to NR database were compared with nRS aligned to NT database to eliminate viral pRS, which correspond to nRS aligning to nonviral nucleotide sequences with a high E-value. To get an idea of the total number of true viral reads belonging to the same virus, the numbers of reads for each RS were eventually summed up. A schematic picture of the bioinformatics analysis scheme is presented in Figure 3.

## 3. Results and Discussion

A panel of 45 primers including 23 forward and 22 reverse primers was designed using the developed algorithm and was synthesized accordingly (Tables 1 and 2). A preliminary analysis using the available control samples (Table 1) demonstrated the ability of the designed primers to perform targeted cDNA enrichment in both single-plex and multiplex formats. This was confirmed by the presence of specific bands of expected lengths in electrophoresis, followed by capillary sequencing (Figures 1 and 2).

**Figure 4:**
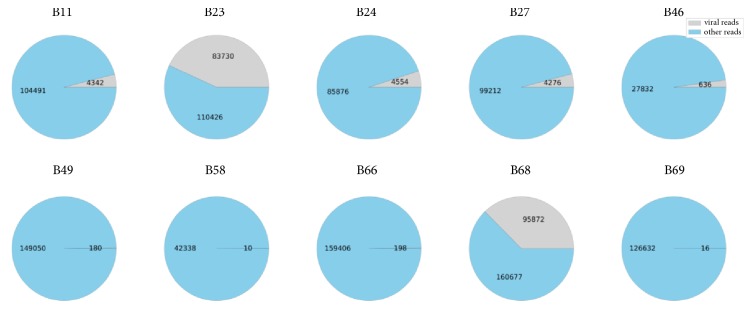
A graphical representation of the percentage of the detected viral reads (grey) with respect to the total number of sequencing reads obtained for the samples listed in Table 3.

Subsequently, we deployed the primer panel to study 70 bird samples (as described in the Methods) and the sequencing results are presented in Table 3 and Figure 4. Only those samples, for which at least 10 viral reads (filtered by quality and length) were identified in the final FASTQ files, are presented in the table. The total number of sequencing reads in such samples is also given to exemplify the percentage of pathogen reads. The alignment of sequencing reads with viral reference sequences was visually inspected to eliminate most of the potential artifacts (such as primer-dimers).

As evident from Table 3, the genera of viruses (samples* B11*,* B23*, and* B24*, highlighted in bold) having a specific primer pair in the panel showed significant amplification, and percentage of the corresponding viral reads ranged between 4.3% and 43.1%. This confirms that our primer panel can efficiently enrich the target genera. Other sequencing reads belonged to bacteriophages, bacteria, and host species. However, we also observed a significant enrichment in the samples* B27* and* B68*, for which a significant number of reads corresponding to Sanxia water strider virus and Duck adenovirus were, respectively, detected. We scrutinized these results in further detail by carrying out a BLAST analysis of all possible versions of the primers (since they are very degenerated) against the sequencing reads and observed that these findings were very likely due to nonspecific amplification from the primers designed for other genera (*gammacorona1_f* and* flavi_r*), though we have not checked this by a direct experiment with a panel that lacks these primers. Although nonspecific amplification allows an even more exhaustive search, it also introduces potentially undesirable enrichment. This could be a problem, especially when the lengths of obtained amplicons are outside the standard intervals suitable for sequencing on most popular platforms.

We also observed a considerable number of viral reads corresponding to various genera in some samples (*B27*,* B46*,* B49*,* B58*,* B66*, and* B69*), despite the absence of specific primers in the panel for their enrichment. The number of reads varied from 0.00013% (Tunis virus) to 2.2% (Watercress white vein virus). The latter is a plant virus and its presence is expected as the feces of the host birds mainly contained grass. These results are possibly due to a weak, nonspecific PCR amplification.

We then checked whether the obtained viral reads for samples* B11*,* B23*,* B24, B27*, and* B68* were due to the amplification with the designed primers in the panel. These five samples were chosen as they indicated the most significant amplification, with 4.0–43.1% of total sequencing reads in the FASTQ files being viral. This was done by repeating a BLAST analysis of all possible versions of the primer sequences against the obtained viral reads for the aforementioned samples and calculating the percentage of those containing at least one primer. We observed that over 99% of the sequences contained the primers validating the effectiveness of the panel.

Finally, in order to confirm the effectiveness of the panel's enrichment, we selected two samples (*B23* and* B66*) for which the presence of viral RNA was shown using amplicon sequencing data (see Table 3). Notably, the* B23* possessed a significant number of reads that correspond to coronaviruses (43.1%) and our panel had specific primers for these genera. As for the* B66* sample, it had a small number of reads that correspond to adenoviruses (0.1%) but we did not design oligonucleotides for them. We then prepared total RNA-seq libraries for these samples, and subsequently sequenced them, yielding ~4.5 million of reads per sample. The percentage of the* coronavirus* reads in the* B23* sample was found to be significantly lower (0.2%) than that in the same sample that was previously enriched by the panel (Table 4). As for the* B66* sample, we identified that 0.05% of reads belong to the* Adenoviridae* family, and most of them were identified as being similar to the Duck and Psittacine adenoviruses. This is similar to the percentage of reads found in the* B66* FASTQ file (0.1%) when the primer panel was applied, i.e., no noticeable enrichment was observed as expected. At the same time, we lost ~0.2% of adenoviruses in the* B23*, but since the amount of data was very different in the two experiments, there was a significant amplification of coronaviruses with almost a half of the reads belonging to these genera; this outcome is not totally surprising. Thus, our approach allows a significant reduction in the cost of sequencing. Besides, for a successful identification of viruses in a preenriched sample, 100,000–200,000 reads per sample are generally enough.

## 4. Conclusions

In this study, we have presented a method of oligonucleotide design for the enrichment of viral nucleic acids. We used this method to design a primer panel, which showed a high efficiency in the detection and identification of several viruses using NGS sequencing. This is especially important for the detection of viruses known to persist in natural zoonotic reservoirs including birds, bats, and rodents, those with a high genetic diversity, and those which can be potentially dangerous to humans and animals. These factors make the development of rapid test-systems essential for the effective management of potential epidemics.

The described method can be recommended to the researchers investigating diverse viromes across different types of biological samples. We deployed our panel for the screening of a number of bird samples and demonstrated a great efficiency. In future studies, the panel will be expanded to target more diverse viruses and tested with bird samples from other regions.

## Figures and Tables

**Table 1:** Viral families and genera covered by the primer panel and control samples of viral RNA used.

**Primer set**	**Family**	***Genus***	**Control sample RNA**	**Acronym**
1	Filoviridae	*Ebolavirus*	Zaire ebolavirus	ZEBOV
2		*Marburgvirus*	Marburg virus	MARV
3		*Cuevavirus*	N/A	

4	Arenoviridae	*Mammarenavirus*	Lassa virus	LASV

5	Rhabdoviridae	*Lyssavirus*	Rabies virus	RABV
6		*Ledantevirus*	N/A	

7	Coronaviridae	*Alphacoronavirus*	N/A	
8		*Betacoronavirus*	Middle East respiratory syndrome coronavirus	MERS CoV
9		*Gammacoronavirus*	N/A	

10	Reoviridae	*Orbivirus*	Kemerovo virus	KEMV
11		*Orthoreovirus*	N/A	
12		*Seadornavirus*	N/A	

13	Paramyxoviridae	*Henipavirus*	N/A	

14	Phenuiviridae	*Phlebovirus*	Uukuniemi virus N/A	

15	Hantaviridae	*Hantavirus*	Dobrava virus	DOBV

16	Nairoviridae	*Orthonairovirus*	Crimean-Congo virus hemorrhagic fever virus, Paramushir virus	CCHFV, PRMV

17	Peribunyaviridae	*Orthobunyavirus*	Inkoo virus	INKV

18	Orthomyxoviridae	*Thogotovirus*	N/A	

19	Picornaviridae	*Cardiovirus*	N/A	
20		*Parechovirus*	N/A	

21	Flaviviridae	*Flavivirus*	Tick-borne encephalitis virus, Yellow fever virus, Japanese encephalitis virus	TBEV, YFV, JEV

22	Togaviridae	*Alphavirus*	N/A	

**Table 2:** Primers used in the panel along with their structures.

**Primer set**	**Primer name**	**Sequence 5'-3'**
1	ebola_f	GCAATGTTCAAACACTTTGTGARGC
	ebola_r	CTTAACACCATAGCAACGGTTR

2	marburg_f	TGGACGATAGGAAATCGAGCAC
	marburg_r	TGAACTATRTTGCCTGAGTAGTGWG

3	cueva_f	GTGCCAGAACAGTTTGAACTCA
	cueva_r	CCGAATTCTCTGGGTAACACAA

4	mammarena_f	CAATMCTTGAYATGGGWCARGG
	mammarena_r	WGATTTRAACTCTGCAACAAAYCTR

5	lyssa_f	CTKGAYTATGARAARTGGAACA
	lyssa_r	TATGTCGGRCAYARAACCTGRT

6	ledante_f	AAYAATACATGGCCCACWCC
	ledante_r	ARTATTCTCTMARMKCCCARGACAT

7	alphacorona_f	GGYACHACHTCWGGTGATGS
	alphacorona_r	GYTTACGYARRTAACCAWAAWABTC

8	betacorona_f	GTGCWAAGAATAGAGCYCGCAC
	betacorona_r	RTCACAYTTWGGRTAATCCCAACCC

9	gammacorona_f	CCACATCTGCTAATGTTGCR
	gammacorona_r	CAGAAATRTCWGCTACAAGACCYTG

10	orbi_f1	TACCGCARGATMGWATGATGAT
	orbi_f2	TATGTTCCWCARGATCGRATGATG
	orbi_r	TGCGCTCCAWAVCCATTCCA

11	orthoreo_f	GTYTCGGCGCCYCAYACDYT
	orthoreo_r	GCAGTRTGCTCAGTDGARGT

12	seadorna_f	CCRCATGAYGTHATGGCYCC
	seadorna_r	TCACCWGACTTAACWCCWGM

13	henipa_f	GGTCAGARACWYTGGTGGAYGA
	henipa_r	ARTAYGGATCACTRGCCCARTC

14	phlebo_f	GATTYAATCTSTKSARRGCY
	phlebo_r	YTATYWGYTCCAYCCAGTYYTC

15	hanta_f	GCWGATGCAACWAARTGGTC
	hanta_r	YARRTTYCCYTGYARCCART

16	nairo_f	CCTTCTTTTSHGGYATGATGCA
	nairo_r	GAAGTTAACACTGNCGAWGTWGCATG

17	orthobunya_f	CWGAWGARATGATWWSTGARCCWGG
	orthobunya_r	GCACTCCATTTWGACATRTCWG

18	thogoto_f	ATCAARGAYMRRCTGAARAANA
	thogoto_r	TCGATSYGMGGCTTTATDGM

19	cardio_f	MRGGYATGGAYCCMATGGAV
	cardio_r	AAGTTRGARTARTCYACATCRTAGA

20	parecho_f	GGRATYAACCCATAYAARGAYTGGC
	parecho_r	GAYCCTGATGGCATACCRCC

21	flavi_f	CTSCTKTGTGACATMGGDGA
	flavi_r	TACATCTCRTGYGTGGARTTBC

22	alpha_f	ACWCTGTTTGTSAACACWGTVRTYA
	alpha_r	CTYTTYARRGGGTCTGCSACHC

**Table 3:** Results of the bird samples sequencing with prior enrichment using the primers panel. The number of viral reads and total number of reads per corresponding sample are shown. For three samples (*B11*, *B23*, and *B24,* highlighted in bold) there was a specific primer pair in the panel present for the amplification of the detected genus. Closest viral homologs names and their GenBank IDs are shown.

**Bird ID**	**Viruses (closest homologs) detected**	**Host** **specie**	**GenBank IDs of the detected viruses**	**Number of viral reads in the sample**	**Total number of reads obtained for the sample**
***B11***	**Duck-dominant coronavirus**∣**Avian coronavirus**∣**Bird droppings coronavirus **	**Taiga Bean Goose**	**AKQ98474.1, AKQ98475.1, APU51837.1, AIY51827.1, CAH69463.1**	**4,342**	**108,833**

***B23***	**Duck-dominant coronavirus∣Avian coronavirus**∣**Bird droppings coronavirus**	**Taiga Bean Goose**	**AKQ98474.1, AKQ98475.1, APU51837.1, AIY51827.1, CAH69463.1**	**83,730**	**194,156**

***B24***	**Duck-dominant coronavirus **	**Taiga Bean Goose**	**AKQ98474.1, AKQ98475.1**	**4,554**	**90,430**

*B27*	Sanxia water strider virus 16	Temminck's Stint	YP_009337377.1	4,062	103,488

*B27 *	Fowl aviadenovirus C∣Turkey aviadenovirus B	Temminck's Stint	ACL68145.1, ANB27700.1, ALY06332.1, ALY06333.1	54	103,488

*B27 *	Cimodo virus	Temminck's Stint	YP_009059075.1	160	103,488

*B46 *	Watercress white vein virus ∣ Turnip yellow mosaic virus	Taiga Bean Goose	AFC95826.1, AMH40128.1	636	28,468

*B49*	Circovirus	Taiga Bean Goose	AEL87792.1	180	149,230

*B58 *	Lake Sarah-associated circular virus-32	Taiga Bean Goose	ALE29729.1	10	42,348

*B66 *	Duck aviadenovirus B	Fieldfare	YP_009047166.1	198	159,604

*B68 *	Duck adenovirus A	Common tern	AGS11269.1, NP_044717.1	95,872	256,549

*B69 *	Tunis virus	Taiga Bean Goose	AMT75434.1	16	126,648

**Table 4:** Comparison of the sequencing results of two samples with and without preenrichment by the primer's panel. In the latter case, the total number of reads was ~4.5 million per sample.

	***B23***		***B66***	
	With enrichment	Total RNA	With enrichment	Total RNA
*Coronaviruses*	43.1%	0.2%	-	-
*Adenoviruses*	-	0.2%	0.1%	0.05%

## Data Availability

All the FASTQ files used to support the findings of this study are available from the corresponding author upon request (Kamil Khafizov; kkhafizov@gmail.com).

## Supplementary Materials

Supplementary Materials
**Table** **S1**: mean, median, and 10th and 90th percentiles of read lengths distributions for unfiltered FASTQ files.
