# Supplementary material for: The Study of Viral RNA Diversity in Bird Samples Using De Novo Designed Multiplex Genus-Specific Primer Panels
Source: Adv Virol. 2018 Aug 12;2018:3248285. doi: 10.1155/2018/3248285 (PMC6109506; doi:10.1155/2018/3248285)
Supplement: Supplementary Materials — Table S1: mean, median, and 10th and 90th percentiles of read lengths distributions for unfiltered FASTQ files. [file 3248285.f1.docx]

**Table S1**. Mean, median, 10^th^ and 90^th^ percentiles of read lengths distributions for unfiltered FASTQ files

| **Bird ID** | **Mean** | **Median** | **10th percentile** | **90th percentile** |
| --- | --- | --- | --- | --- |
| B11 | 158 | 160 | 89 | 230 |
| B23 | 185 | 222 | 96 | 227 |
| B24 | 133 | 118 | 73 | 223 |
| B27 | 145 | 160 | 57 | 218 |
| B46 | 106 | 97 | 50 | 198 |
| B49 | 166 | 153 | 93 | 288 |
| B58 | 123 | 107 | 54 | 227 |
| B66 | 165 | 166 | 94 | 245 |
| B68 | 201 | 230 | 106 | 254 |
| B69 | 163 | 179 | 90 | 231 |
